# Massachusetts Dental Society

**Published:** 1900-01

**Authors:** James H. Daly

**Affiliations:** Boston, Mass.


### MASSACHUSETTS DENTAL SOCIETY.

(Continued from Vol. XX., page 822.)

### THE SIXTH-YEAR MOLAR.

BY JAMES H. DALY, D.D.S., BOSTON, MASS.

THERE is perhaps nothing with which the dental profession has concern which is of more profound interest and importance than those teeth which are described as the sixth-year molars, or the first permanent molars. There is nothing which so often and so surely leads to misadventure than the lack of information which pervades the people at large concerning these peculiar but important teeth. This lack of information pervades all classes,—the most ignorant and, in other matters, the most learned. Perhaps the most common experience of every member of our profession is the insistence of parents that a refractory sixth-year molar is a "first tooth," and hence that no harm can befall the child from its sacrifice. A "first tooth" the sixth-year molar undoubtedly is, but it is not to be classed with the deciduous teeth, and, in place of the almost universal lack of care for its preservation, let us hope that a complete knowledge of its true character and importance may come to the mass of the people. This accomplished, one of the most potent causes of perplexity in our profession will be eliminated.

It is important that we should impress upon the minds of our patients, especially those who are parents, the fact that these teeth, although they are of early growth, possess many of the qualities which characterize the deciduous teeth. They are often faulty in structure, imperfectly developed, and liable to be attacked by caries. Often there are small indentations or fissures in the enamel and on the grinding surface, leaving the dentine exposed to decay. Often they are attacked by caries almost immediately after they have erupted, and decay in them is often exceedingly rapid. But, notwithstanding these peculiar characteristics, these molars are the largest teeth in the mouth and have the largest roots, and, if they have escaped the defects noticed, they are often the strongest teeth and of great value.

The sixth-year molar usually makes its appearance at a period of life between the fifth and the seventh year. It has been my experience that these teeth are seen most frequently after the age of five and one-half years. The statement of a writer in "American System of Dentistry," that these teeth are often seen shortly after the fourth year, I believe to be erroneous. The eruption at this early age—five and one-half to seven years—causes them to be mistaken for temporary or deciduous teeth, and hence the appearance of decay causes no alarm upon the part of parents, and they are allowed to suffer neglect. The child's first visit to the dentist is more frequently than otherwise occasioned by pain in one of these teeth, which has thus been allowed to decay without attempt at preservation. When this occurs the decision of the parent is almost invariably for a removal of the offending tooth.

Dr. Eugene S. Talbot, in a dissertation entitled "A Study of the Degeneracy of the Jaws of the Human Race," says that in England and on the Continent it is thought the proper thing to extract the sixth-year molars as deciduous teeth; and in reply to a letter addressed to a Glasgow dentist, asking for assistance in making examination of the mouths of patients, the latter said, "I am sorry to say that I am unable to procure the measurements you desire, as it is rare, indeed, to find the sixth-year molars in position in persons of twenty-five years of age, especially those of the Scotch people." At times the mischief has so far advanced in these teeth that extraction is inevitable. But the careful and thoughtful practitioner urges that between the seventh and eighth year the extraction of the teeth should, if possible, be avoided. And if this act is performed after the emergence of the twelfth-year molars the result is positively disastrous. Unless the quality and conditions of these teeth is such as to render their preservation impossible, or a positive menace to health, they should be given the benefit of the utmost art of the dentist for their preservation at least until a period just before the eruption of the twelfth-year molars. If this can be done the second molars will come forward and take the places of those extracted.

"The title of the sixth-year molar to longevity," says Dr. Weld, "can only be questioned under neglect and abuse. It is the keystone molar: with it the integrity of the arch is preserved; without it the usefulness of the arch is impaired, if not destroyed. Its extraction at an early age signifies a loss of masticating surface that is absolutely detrimental to the health and comfort of the patient. In view of the prominent position it occupies in the arch, and its relation and influence as a just poise or balance in the distribution of the varied strains incident to mastication, its extraction can be considered a physiological mistake."

These molars appear at a period in the life of a child when he requires gentle handling. Their loss often means far more than the loss of an individual tooth, for by this means the entire mouth is often crippled. The grinding surface is frequently greatly impaired, correct occlusion is rendered impossible, and the entire arrangement of the jaw is not infrequently disorganized. The habit of extracting these molars, pursued from generation to generation, will, in the course of time, cause an inherited smallness of jaw. The error of this custom, which prevails too much in this country as well as in Europe, is apparent to the thoughtful practitioner. He cannot fail to observe that, when these teeth are removed at a point of time when the arrival of the second molars will not effect a partial substitution, the effort of nature to bridge the space thus formed often produces a condition lifelong in its discomfort and inefficiency.

Is it possible, then, that these valuable teeth can be preserved and the unity of the oral arch maintained? Difficulties obviously intervene, but difficulties which are not insurmountable. The first of these difficulties is undoubtedly that which has already been considered,—the lack of popular knowledge upon this topic. Could the truth be impressed upon the minds of parents that the molars which appear in the mouth of the child at about the sixth year are designed by nature to be not temporary but permanent teeth, much of the difficulty would be surmounted. This knowledge gained, the parent would not only seek the aid of the dentist upon the first appearance of caries, but would also cheerfully acquiesce in the adoption of any method suggested for the preservation of these teeth. Not only these, but with the possession of knowledge concerning the permanent value of these teeth, parents would not neglect the care of the diet of their children, to the end that these teeth, in their formative period, will receive such nourishment as may be best suited to growth. "The first molars are developed," says Dr. Marshall, in the *Dental Practitioner*, "at a period when the demand for bone-producing elements in the system is greater in proportion to the supply than at any other, and unless these elements are forthcoming in sufficient quantity the teeth are sure to suffer, since nature supplies first those parts which are of more vital necessity,—the bones of the skeleton. We as a people are proud to eliminate from our food products those parts in which are stored, in greatest quantities, the elements so necessary in the formation of good enamel and dentine. Children should be encouraged to eat such food as contains the bone-producing elements in the best proportions, and in the best form for ready assimilation, prepared in such a manner as to require thorough mastication."

Such increased intelligence and co-operation on the part of parents being gained, the problem of the preservation of the first permanent molars is more than half solved. When, however, the patient is brought, and evidence of more or less advanced decay in these teeth is apparent, *the work of the operator* is before him. Having first persuaded the parent of the necessity for the preservation of the affected teeth, the best method for the case in hand should be applied. It is no part of the plan of this paper to instruct in methods. It may be suggested, however, that such teeth, if not too far gone by decay, may be preserved by temporary stopping. Further than this the judgment of the operator in each case must be exercised. Extraction should be resorted to only when it becomes improbable that, at eleven years of age, these teeth will survive for any considerable length of time. When the pulp is devitalized, before proper formation of the root structure, when a protrusion of either arch or other irregularity or false occlusion may be corrected by removal, then only is it proper to apply the forceps.

The thought to be enforced chiefly by this paper is that of the urgent necessity for a diffusion of common knowledge upon this important point. The suggestion seems not inapt that to this end concerted action should be taken by dental societies, in what may be properly called a "campaign of education." The publication of a pamphlet for distribution among the schools and for general distribution in the homes—a pamphlet which would not bear the external aspect of an advertisement—might perhaps accomplish much in the attainment of this much-desired end.

DISCUSSION OF DR. DALY'S PAPER.

*President Draper.*—This subject seems to recur periodically, and it is one that seems will never be settled. In the *Dental Cosmos* I notice a letter from Dr. Mitchell, of London, advocating the removal of the sixth-year molars in many cases. The paper is now open for discussion, and it is to be hoped we will hear from both sides of the question.

*Dr. William O. Barrett* (Ware, Mass.).—Children of about eight years come to my office with the sixth-year molars in such a wretched condition that it seems absolutely necessary to extract them. And still the extracting of these molars is exceedingly unfortunate when the temporary molars have been lost, as it leaves the little patient with only the front teeth to masticate its food until the bicuspids take their occluding positions.

I have a case in my own family where the early extraction of the sixth-year molars has caused the twelfth-year molars to tip forward, and so badly expose the pericementum on the posterior surface. The wisdom-teeth crowd upon these exposed parts of the root and invite caries right at the neck of the twelfth-year molars. These cavities are not only very inaccessible and sensitive, but even after they are filled the position of the teeth produces such unfavorable hygienic conditions that these fillings are very likely to fail.

The paper emphasizes a valuable point where it speaks of the importance of teaching the care of the teeth in our public schools.

*Dr. H. A. Baker* (Boston, Mass.).—I have not had the experience or opportunities of a friend of mine, whom I would like to hear speak on this subject. I refer to Dr. Bogue, of New York.

*Dr. E. A. Bogue* (New York, N. Y.).—I am to have the honor of saying something on this subject indirectly this evening. It is hardly worth while, therefore, to say anything at this time; but I am compelled to express my thanks to Dr. Daly for what he has said and to express my regrets that he was not more emphatic. I can express my feelings by speaking of two cases. A young lady of twelve years of age came to me with no sixth-year molars. The extractions were done by advice of an educated gentleman. I had to put in a plate for that child to eat upon while the twelfth-year molars were being developed. She was ill from inability to masticate.

In the second case, the daughter of a foreign minister, sixteen years of age, the left lower molar was abscessed and so badly decayed that the two roots were separated. The right lower molar was abscessed and the crown half gone, while the left upper molar was nearly half gone and abscessed, and the right one had an exposed pulp.

Now, if any one ever had an excuse for extracting the sixth-year molars, I think I had in that case; but I recalled some sad mistakes I had made in previous years, when by extracting I had done more harm than good, as was developed by the passage of some years.

Fearing the results that were sure to arise from extracting, I treated both of the abscessed lower molars, and when the separated roots were well, I inserted a screw into each root strongly; then covering the exposed gums between the roots with gutta-percha, I put a platinized gold ring around the roots on the left-hand side and filled it with amalgam. The other lower molar on the right was filled with a ring around it, and the upper molars were restored to health and their normal shape by filling with amalgam.

The young lady's health began at once to improve, and she regained her vigor, married, and has now quite a family.

*Dr. C. A. Lindstrom* (Lynn, Mass.).—I would like to ask Dr. Bogue how long ago this work was done, and how long, or what will be the condition you anticipate in a few years from now, in the case of the second teeth that were filled with amalgam after they were abscessed?

*Dr. E. A. Bogue.*—This was done about eight years ago.

*Dr. C. A. Lindstrom.*—What condition did you find them in, if you have seen them lately?

*Dr. E. A. Bogue.*—I have not seen them lately.

*Dr. C. H. Davis* (Worcester).—Do you know to what extent the food or other substances may have crowded underneath the crown and caused the roots to loosen?

*Dr. E. A. Bogue.*—No, I do not.

*Dr. C. H. Davis.*—I did a similar thing some seven or eight years ago, and put on a gold crown where the two roots were entirely separated and the gums grown over. I crowned each root separately, then put a crown over that, and put on the bridge. This bridge has done remarkable service.

*Dr. Edwin E. Davis* (Boston, Mass.).—I had a case which bears a little on this paper, where the crown had decayed, leaving the roots separated as mentioned. After treating the roots, I adjusted a gold crown to each root, treating each root as a separate bicuspid root. The bite was extremely short, and brought no extra strain upon either root. This was very effective, and at last accounts was in good condition.

*Dr. G. A. Maxfield.*—What was the age of the patient?

*Dr. Edwin E. Davis.*—At the time of treatment the patient was eight or nine years old.

*Dr. W. O. Barrett.*—If the sixth-year molar is to be extracted, it should be done between the eighth and ninth years, before the twelfth-year molar is ready to appear.

*Dr. J. H. Daily* (Boston).—If the sixth-year molar is to be extracted, it should *not* be done between the eighth and ninth years, but *just before* the twelfth-year molar is ready to appear, or at the eleventh year. Over twenty years ago I took a young patient to a well-known practitioner in Boston, and he advised the extraction of the sixth-year molars. I took out four teeth, and injured him for life. Every tooth separated; not a single tooth backed up its neighbor. That poor miserable root which you are obliged to labor over in order to save, and which at times seems almost beyond hope, is worth all the time, labor, and trouble which it is necessary to put into the work; every year that root is saved adds just so much to the appearance and good condition of the other teeth. They will not tip over in later years, and every year that passes makes each individual tooth more independent of its neighbor.

I feel that I have done that young lad an injury I can never undo. Every tooth has taken since then a more or less abnormal appearance. I must question if they ever would have decayed if they had been separated by one another. The tooth that is next its neighbor, supported by its proper neighbor, is very much less likely to decay.

*Dr.* ——.—The extraction of the sixth-year molar surely causes a great deal of trouble, but this is not always the case. I know of a case where three were taken out and one kept. Where the three were taken out everything was all right; where the one was left the bicuspids were broken down.

*Dr. E. A. Bogue.*—I will present five hundred dollars to any one who will give me, from any source whatever, one single case where the articulation of the teeth is perfect after the loss of one single tooth.

*Dr. Geo. A. Maxfield.*—Every one of us present realizes the necessity of saving the first molars. The question that troubles most of us is that the patient has not reached that stage of development where he can see the advantage that can be gained by saving the teeth. We have that experience every week. It is often difficult to control some patients. The cavity is in such shape that we cannot clean it out to relieve the pain, and the next thing is the tooth must come out. Last week a father came in with his child, and demanded that I extract the tooth that I was trying to save. I refused, and have lost that family as a result. I do not like to extract a tooth for a child, but it is a question what is just the right thing to do.

I have seen cases where the first molar has been removed and the second has found its place. I also have seen a good many mouths with all the teeth in, and in these the articulation is not perfect. I would not detract from anything that Dr. Bogue has said, because it is scientifically correct.

*Dr. Carl R. Lindstrom.*—What can we do with the many cases which come to us in practice where patients are not able either to give the time or to incur the expense of treatment? Now, dentists are not always able to render gratuitous services; they can do so sometimes; but in my practice, and I think it is so with many, what shall we do when these patients come to us and these teeth can be saved? Sometimes we may succeed in bridging over a period of life, but at other times we cannot do so. Now, what is the proper procedure? I have seen in my own practice many mouths where the sixth-year molars have been extracted, and which were in good condition. It seems to me, although I recognize the truth of what Dr. Bogue says, that we must recognize the truth on the other side. Dr. Maxfield sent his patient away simply to have him go to some one else. If that patient could not afford proper treatment, and Dr. Maxfield could not afford to take him for a charity patient, somebody else had to extract that tooth. Now, I would like to hear from the other side what should be done in such cases.

*Dr. N. Morgan* (Springfield).—I wish we could have our old friend Dr. Riggs with us for a few moments to give us his experience in this branch. I have had failures in results of removing these teeth, and also many which I esteemed successful. There are many things to consider in these cases. The treating of badly broken down sixth-year molars for children seven to fourteen years of age is a serious matter. In children of well-to-do parents there is frequently a lack of vital force in their constitutions, which makes these difficult operations almost impossible. The children of the poorer class are more inured to hardships and will endure the operations better; but the expense cannot well be afforded, and we do not care for too many charity patients. It seems to me that each case must have individual study and the treatment such as is possible with the child, and as far as may be for its future welfare and within the parents' means.

*Dr. R. H. Clark.*—Accepting the fact that these teeth should be saved wherever it is advisable, there is one question that I should like to have answered to my satisfaction by those who have had more experience in root-canal fillings than I have. Some children have roots developed quicker than others, some children of eight years have roots developed, and others have not; but with a child who has a permanent tooth that needs attention and is willing to have attention given it, and we find upon close examination that the root is not thoroughly developed, what kind of root filling are we going to place into that tooth? Some root fillings are irritating and cause a bad effect. What is the best filling in such cases as these?

*Dr. C. H. Davis* (Worcester, Mass.). In this line I have had quite a little experience, and very much to my sorrow it has been with my own child. When I was six years old I came in contact with a horse's foot; I had my jaw broken and my nose broken, and lost some of my second teeth. I have them in a bottle in my office, and they have been a great source of instruction and very useful in demonstrating that certain kinds of work could not be done upon children's teeth. I had a young girl come to me with quite a prominent upper jaw. She had fallen on the sidewalk at ten years old and had broken both her central incisors. I advised putting some porcelain tips on both of them, the parents were willing that this should be done, but wanted to consult with other dentists in the city. This I was agreeable to, and after consultation with some of the prominent practitioners they agreed with me as to how the work should be done, and I did the work. Soon after, the girl's mother came to me and wanted to know why I had not crowned those teeth. Dr. So-and-So said it could be done for fifteen dollars (which was much less than my charge), and look a great deal better than they did with porcelain tips. I said to her, "Do you know the condition your daughter's teeth are in at her age?" She said, "Why?" I took out my little bottle with five or six of my teeth in it and showed her the teeth that were broken off (the central incisors), calling her attention to the root development. If I had cut them for crowns, as the other dentist would have done, what would I have had to put the crown upon? I would have had a short stump with a large hole in it, and nothing to hold the crown firmly. I put those tips on nearly ten years ago, and did some work for the lady last week. Those tips were in good condition.

My boy had his teeth broken off (the central incisors) when he was six and a half years old. He was petting a cow, and as the flies were troublesome she threw her head, striking him with her horn and breaking the teeth. I could not do much for him at that time, but when I came to work into that canal I found it was in the same condition as the one which I have described. I took a little ball of gutta-percha, dipped it in chloroform to soften, measured the length of the canal, and with warm instruments packed carefully the canal. It never gave any trouble whatever. I kept the tooth built down with cement for several years, with a staple in the canal for anchorage. He is now nearly twelve. It has been on five years, and the root is perfectly solid to-day.

Last winter, finding the root firm after being filled some five years, I put a Richmond crown upon it. I was able to do this, as the tooth was but partially erupted, giving me, in addition to the short root, about one-half of the crown of the tooth to hold the pin and band of the Richmond crown, and thus far it has given no trouble.

(To be continued.)

